# *In vitro* antimicrobial susceptibility of clinical isolates from adult and paediatric patients in Jordan: Antimicrobial Testing Leadership and Surveillance (ATLAS) 2010–2021

**DOI:** 10.3389/frabi.2024.1375980

**Published:** 2024-08-08

**Authors:** Dima Al Jammal, Julia Bachir, Jihane A. Moussa, Jamal Wadi Al Ramahi

**Affiliations:** ^1^ The Medical School, University of Jordan, Amman, Jordan; ^2^ Pfizer Inc., Medical Affairs, Beirut, Lebanon

**Keywords:** antimicrobial resistance, antimicrobial surveillance, clinical isolates, beta-lactamases, phenotypes, Jordan

## Abstract

**Objectives:**

To evaluate the *in vitro* antimicrobial susceptibilities of Gram-positive and Gram-negative isolates from patients in Jordan between 2010 and 2021, through the Antimicrobial Testing Leadership and Surveillance (ATLAS) programme.

**Methods:**

Medical centres in Jordan collected bacterial isolates from hospitalised patients with defined infection sources between 2010 and 2021 (no isolates collected in 2014). Antimicrobial susceptibility was interpreted using CLSI standards. FDA-approved breakpoints were applied for tigecycline. The identification of β-lactamase genes was performed for a proportion of isolates using multiplex PCR assays.

**Results:**

More than 92% of *Acinetobacter baumannii* collected were multidrug-resistant (MDR) and/or carbapenem-resistant (CR), and > 50% susceptibility was reported only to minocycline (62.2% among both MDR and CR isolates). Rates of MDR and CR *Pseudomonas aeruginosa* were 14.3% and 20.5%, respectively, and among all *P. aeruginosa* collected from adults, susceptibility to ceftazidime/avibactam was 95.3% and to ceftolozane/tazobactam was 88.4%. For *Escherichia coli* from adults and MDR *E. coli*, susceptibility to ceftazidime/avibactam, ceftolozane/tazobactam, imipenem, meropenem and meropenem/vaborbactam was 92.1%–98.7%. Susceptibility to tigecycline was > 94% among *Klebsiella pneumoniae* from adult, paediatric, and ICU patients (all ages). CTX-M-15 was the most frequently identified β-lactamase gene among *E. coli* and *K. pneumoniae*. Susceptibility to most antimicrobial agents was < 50% among *K. pneumoniae* carrying CTX-M-15, CTX-M-9-type, NDM-5, and/or OXA-48 β-lactamase genes. All *S. aureus* collected were susceptible to teicoplanin, vancomycin, daptomycin, linezolid and tigecycline, with 96.1% of *S. aureus* from adults were susceptible to ceftaroline. Overall, 58.8% of *Staphylococcus aureus* were MRSA.

**Conclusion:**

This study provides valuable information regarding antimicrobial susceptibility in Jordan between 2010 and 2021. Continued monitoring of *in vitro* antimicrobial susceptibility is critical in the fight against antimicrobial resistance.

## 1 Introduction

The burden of antimicrobial resistance is well-documented, with the World Health Organization naming it among the top 10 global public health threats (WHO, 2021). Antimicrobial resistance was estimated to be associated with nearly 5 million deaths worldwide in 2019, among which *Escherichia coli*, *Staphylococcus aureus*, *Klebsiella pneumoniae*, *Streptococcus pneumoniae*, *Acinetobacter baumannii* and *Pseudomonas aeruginosa* were responsible for 72.1% (Antimicrobial Resistance Collaborators, 2022). Antimicrobial activity can be limited, or even eradicated, by the resistance mechanisms displayed by these clinical pathogens, with carbapenem-resistant (CR) *A. baumannii* and *P. aeruginosa*, third-generation cephalosporin-resistant *E. coli* and methicillin-resistant *S. aureus* (MRSA), listed as critical or high-priority targets for new antimicrobial agents (Tacconelli et al., 2018).

The surveillance of antimicrobial activity plays a crucial role in efforts to tackle antimicrobial resistance. One such surveillance programme is the Antimicrobial Testing Leadership and Surveillance (ATLAS) database (Antimicrobial Testing Leadership and Surveillance (ATLAS) database, 2023). The ATLAS database was initiated in 2018 and integrates historical data from earlier surveillance programmes, Tigecycline Evaluation and Surveillance Trial (TEST) and International Network for Optimal Resistance Monitoring (INFORM), with regularly updated antimicrobial surveillance data from countries worldwide. The database reports laboratory information collected from clinical Gram-negative and Gram-positive isolates and tracks the antimicrobial activity of a panel of agents, including aztreonam/avibactam, ceftaroline, ceftazidime/avibactam, ceftolozane/tazobactam and meropenem/vaborbactam.

Ceftolozane/tazobactam, ceftazidime/avibactam and meropenem/vaborbactam are all approved for clinical use against antimicrobial-resistant pathogens, and a meta-analysis determined a pooled clinical success rate of 73.3% for all three agents against multidrug-resistant (MDR) Gram-negative organism infections (Wilson et al., 2021). In addition, the combination of aztreonam with avibactam is currently being tested in Phase 3 clinical trials for the treatment of serious bacterial infections caused by Gram-negative pathogens, including MDR metallo-β-lactamase (MBL)-producers (Pfizer Inc, 2023).

Although antimicrobial surveillance data has been frequently published for some geographical regions, there is a paucity of data for others. Therefore, we set out to report on antimicrobial activity and susceptibility among bacterial isolates submitted to ATLAS from centres in Jordan. Previous ATLAS publications have included data from Jordan, presented as part of the Middle East region (Piérard and Stone, 2021; Karlowsky et al., 2021a, 2022; Estabrook et al., 2023; Wise et al., 2023), however, this is the first country-specific ATLAS study on isolates of Gram-negative and Gram-positive pathogens from patients in Jordan. Isolates were collected between 2010 and 2021.

## 2 Materials and methods

### 2.1 Participating centres and bacterial isolates

Two medical centres in Jordan collected bacterial isolates from hospitalised patients between 2010 and 2021 (no isolates were collected in 2014). The ATLAS protocol defined the number of bacterial isolates per species that each centre could submit each year, and also defined the acceptable infection sources (intra-abdominal, urinary tract, skin and skin structure, lower respiratory tract or bloodstream infections). Isolates were consecutive, nonduplicate clinical isolates and limited to one per patient per year. Selected demographic data on patient age, sex and ward location, were also collected and are presented in **Supplementary Table 1**.

### 2.2 Antimicrobial activity testing

Following identification at the local participating centre, all isolates were shipped to a central reference laboratory (International Health Management Associates, Inc. Schaumburg, IL, USA) for species confirmation using MALDI-TOF (Bruker Biotyper MALDI-TOF, Bruker Daltonics, Billerica, MA, USA).

Antimicrobial MIC values were determined using CLSI broth microdilution methodology (CLSI, 2018). MICs for aztreonam/avibactam and ceftazidime/avibactam were determined at a fixed concentration of 4 mg/L for avibactam (CLSI, 2023). Antimicrobial susceptibility was interpreted according to CLSI guidelines, except for tigecycline, where the FDA-approved breakpoints were applied for *S. aureus* (including MRSA), *S. pneumoniae*, *E. faecalis* (vancomycin-susceptible isolates), and the Enterobacterales (CLSI, 2023; FDA, 2023).

### 2.3 Antimicrobial resistance phenotypes

The definitions of the resistance phenotypes presented, determined using CLSI breakpoints, are listed in full on the ATLAS website (Antimicrobial Testing Leadership and Surveillance (ATLAS) Definitions, 2023). CR *A. baumannii* or *P. aeruginosa* were defined as resistant to meropenem. MDR *A. baumannii*, *P. aeruginosa*, *E. coli* or *K. pneumoniae* were defined as resistant to ≥ 3 antimicrobial agents from the ATLAS testing panel (excluding agents to which an organism has intrinsic resistance): aminoglycosides (amikacin or gentamicin), carbapenems (doripenem, ertapenem, imipenem or meropenem), cephalosporins (cefepime, ceftriaxone or ceftazidime), quinolones (ciprofloxacin or levofloxacin), penicillin plus β-lactamase inhibitor combinations (ampicillin/sulbactam, amoxicillin/clavulanate or piperacillin/tazobactam), or polymyxins (colistin). Additional agents to be considered for each organism are listed on the ATLAS website (Antimicrobial Testing Leadership and Surveillance (ATLAS) database, 2023).

### 2.4 Screening for β-lactamase genes

As described previously (Kazmierczak et al., 2018; Piérard and Stone, 2021; Wise et al., 2023), isolates of Enterobacterales with MIC values of ≥ 2 mg/L to meropenem (meropenem-nonsusceptible) (CLSI, 2023), were screened for genes encoding clinically relevant β-lactamases (SHV, TEM, CTX-M, VEB, PER and GES ESBLs; ACC, ACT, CMY, DHA, FOX, MIR and MOX plasmid-mediated AmpC β-lactamases; GES, KPC and OXA-48-like serine carbapenemases; and NDM, IMP, VIM, SPM and GIM MBLs), using published multiplex PCR assays (Lob et al., 2015). Full details of the screening criteria for isolates of Enterobacterales during the study period have been previously published (Kazmierczak et al., 2018; Piérard and Stone, 2021; Wise et al., 2023). From 2020 onwards, not all isolates that qualified for screening were tested for genes encoding relevant β-lactamases. In 2020, 13 of 28 (46.4%) *E. coli* and *K. pneumoniae* isolates from Jordan that qualified for screening were tested for β-lactamase genes and in 2021, 29 of 46 (63.0%) qualifying *E. coli* and *K. pneumoniae* isolates were tested.

All detected carbapenemase genes were amplified using flanking primers and sequenced, and sequences were compared against publicly available databases maintained by the National Center for Biotechnology Information (www.ncbi.nlm.nih.gov) (Estabrook et al., 2023; FDA, 2023). In the present study, MIC summary and susceptibility data for organisms carrying one of the aforementioned resistance genes are presented where ≥ 20 isolates were reported.

## 3 Results

### 3.1 Demographic information

A total of 1,856 isolates (Gram-negative, 67.2%; and Gram-positive, 32.8%) were collected between 2010 and 2021, except for 2014 when no isolates were collected (**Supplementary Table 1**). Most of the total number of isolates were from adults (age ≥ 18 years, 77.9%) and approximately half were from male patients (51.2%). The most common source of isolates was genitourinary (25.8%), followed by circulatory (19.6%) sources. The percentage distribution of the total number of isolates among each source type was comparable (1.1–1.6-fold difference) between the Gram-negative and Gram-positive species, with the most notable difference seen for respiratory sources (Gram-negative, 19.6% and Gram-positive, 8.2%; 2.4-fold difference). Most isolates were from general medicine wards (24.7%), followed by general surgery wards (18.0%). Susceptibility data are presented for *A. baumannii, P. aeruginosa, E. coli, K. pneumoniae, Enterococcus faecalis, S. aureus* and *S. pneumoniae*.

### 3.2 Antimicrobial susceptibility of non-fermenting Gram-negative organisms

#### 3.2.1 *Acinetobacter baumannii* (N = 160)

Among *A. baumannii* isolates from adult patients and ICU patients (all ages), the highest rates of susceptibility were to minocycline (62.6% and 53.3%, respectively) (**Table 1**). For the paediatric subset there were no data for minocycline and the highest rate of susceptibility was to levofloxacin (28.6%).

**Table 1:** *In vitro* activity and antimicrobial susceptibility of the ATLAS antimicrobial panel against clinical isolates of *Acinetobacter baumannii* and *Pseudomonas aeruginosa* collected in Jordan between 2010 and 2021.

	Adult				Paediatric				ICU (all ages)			
		MIC (mg/L)				MIC (mg/L)				MIC (mg/L)		
Organism^a^/Agent^b^	n	90%	Range	%S	n	90%	Range	%S	n	90%	Range	%S
*Acinetobacter baumannii*												
Ampicillin/sulbactam	48	≥128	8–≥128	2.1	–	–	–	–	27	64	16–≥128	0.0
Piperacillin/tazobactam	139	≥256	0.12–≥256	4.3	21	≥256	0.12–≥256	14.3	72	≥256	16–≥256	2.8
Cefepime	139	≥64	2–≥64	5.8	21	≥64	2–≥64	14.3	72	≥64	4–≥64	2.8
Ceftazidime	139	128	2–≥256	5.8	21	≥128	4–≥128	19.0	72	128	4–≥256	4.2
Ceftriaxone	91	64	8–≥128	2.2	–	–	–	–	45	≥128	32–≥128	0.0
Aztreonam/avibactam	48	128	16–128	NA	–	–	–	–	27	128	32–128	NA
Imipenem	48	≥16	2–≥16	2.1	–	–	–	–	27	≥16	8–≥16	0.0
Meropenem	139	≥32	≤0.06–≥32	5.0	21	≥32	0.25–≥32	19.0	72	≥32	1–≥32	1.4
Ciprofloxacin	48	≥8	4–≥8	0.0	–	–	–	–	27	≥8	4–≥8	0.0
Levofloxacin	139	≥16	0.06–≥16	5.0	21	≥16	≤0.25–≥16	28.6	72	≥16	0.25–≥16	2.8
Gentamicin	48	≥32	0.5–≥32	10.4	–	–	–	–	27	≥32	0.5–≥32	18.5
Amikacin	139	≥128	1–≥128	19.4	21	≥128	1–≥128	23.8	72	≥128	2–≥128	16.7
Minocycline	91	16	≤0.5–≥32	62.6	–	–	–	–	45	16	≤0.5–≥32	53.3
Tigecycline	139	2	0.06–8	NA	21	1	0.12–2	NA	72	2	0.25–8	NA
Colistin	48	1	≤0.12–2	0.0	–	–	–	–	27	1	0.25–1	0.0
Trimethoprim/sulfamethoxazole	48	≥64	1–≥64	25.0	–	–	–	–	27	≥64	1–≥64	37.0
*Pseudomonas aeruginosa*												
Piperacillin/tazobactam	170	128	0.25–≥256	76.5	37	32	0.12–≥256	86.5	47	128	2–≥256	70.2
Cefepime	170	16	≤0.5–≥64	78.8	37	16	≤0.5–≥64	78.4	47	32	≤0.5–≥64	74.5
Ceftazidime	170	32	0.5–≥128	81.2	37	16	1–≥64	86.5	47	32	1–≥64	78.7
Ceftazidime/avibactam	64	4	0.25–≥128	95.3	–	–	–	–	-	-	-	-
Ceftolozane/tazobactam	43	8	0.5–≥32	88.4	–	–	–	–	-	-	-	-
Aztreonam	64	32	0.12–64	71.9	–	–	–	–	-	-	-	-
Aztreonam/avibactam	64	16	0.06–64	NA	–	–	–	–	-	-	-	-
Imipenem	64	≥16	0.12–≥16	67.2	–	–	–	–	-	-	-	-
Meropenem	170	16	≤0.06–≥32	70.6	37	16	≤0.06–≥32	62.2	47	16	≤0.06–≥32	53.2
Ciprofloxacin	64	4	≤0.06–≥8	81.3	–	–	–	–	-	-	-	-
Levofloxacin	170	≥16	≤0.25–≥16	63.5	37	≥16	0.06–≥16	73.0	47	≥16	0.25–≥16	61.7
Amikacin	170	32	≤0.5–≥128	87.1	37	16	1–64	91.9	47	32	1–≥128	87.2
Tigecycline	170	16	≤0.008–≥32	NA	37	16	0.5–≥32	NA	47	16	1–≥32	NA
Colistin	64	2	≤0.12–≥16	0.0	–	–	–	–	-	-	-	-

MIC, minimum inhibitory concentration; NA, breakpoints not available; S, susceptible.

-, data not available.

a No isolates were collected in 2014. In addition, there were no data for the following agents in the following years: cefepime, ceftazidime and amikacin in 2019; minocycline in 2018–2021; ampicillin/sulbactam, gentamicin and trimethoprim/sulfamethoxazole in 2010–2017; imipenem, ciprofloxacin, colistin, ceftazidime/avibactam and aztreonam in 2010–2017 and 2019; and ceftolozane/tazobactam in 2010–2019.

b Some antimicrobial agents were tested against fewer isolates due to fewer years of isolate collection.

A total of 149 (93.1%) *A. baumannii* isolates were MDR and 148 (92.5%) were CR (**Table 2**). Susceptibility to minocycline was 62.2% among the MDR and CR subsets, followed by trimethoprim/sulfamethoxazole (29.4% and 30.0%, respectively). The MIC_90_ of tigecycline was 2 mg/L against the adult, ICU, MDR and CR subsets of *A. baumannii* (**Tables 1**, **2**), and for isolates of *A. baumannii* from paediatric patients, the MIC_90_ for tigecycline was 1 mg/L (**Table 1**).

**Table 2:** *In vitro* activity and antimicrobial susceptibility of the ATLAS antimicrobial panel against clinical isolates of *Acinetobacter baumannii* and *Pseudomonas aeruginosa* with resistance phenotypes collected in Jordan between 2010 and 2021. .

	Multidrug-resistant				Carbapenem-resistant			
		MIC (mg/L)				MIC (mg/L)		
Organism^a^/Agent^b^	n	90%	Range	%S	n	90%	Range	%S
*Acinetobacter baumannii*								
Ampicillin/sulbactam	51	64	8–≥128	2.0	50	64	16–≥128	0.0
Piperacillin/tazobactam	149	≥256	64–≥256	0.0	148	≥256	16–≥256	0.7
Ceftriaxone	98	≥128	64–≥128	0.0	98	≥128	32–≥128	0.0
Cefepime	149	≥64	8–≥64	0.7	148	≥64	4–≥64	1.4
Ceftazidime	149	128	4–≥256	1.3	148	128	4–≥256	2.0
Aztreonam/avibactam	51	128	16–128	NA	50	128	32–128	NA
Imipenem	51	≥16	2–≥16	2.0	–	–	–	–
Meropenem	149	≥32	≤0.06–≥32	1.3	–	–	–	–
Ciprofloxacin	51	≥8	4–≥8	0.0	50	≥8	4–≥8	0.0
Levofloxacin	149	≥16	0.25–≥16	1.3	148	≥16	0.25–≥16	2.0
Amikacin	149	≥128	1–≥128	14.1	148	≥128	1–≥128	14.9
Gentamicin	51	≥32	0.5–≥32	9.8	50	≥32	0.5–≥32	10.0
Colistin	51	1	≤0.12–2	0.0	50	1	0.25–2	0.0
Minocycline	98	16	≤0.5–≥32	62.2	98	16	≤0.5–≥32	62.2
Tigecycline	149	2	0.06–8	NA	148	2	0.06–8	NA
Trimethoprim/sulfamethoxazole	51	≥64	1–≥64	29.4	50	≥64	1–≥64	30.0
*Pseudomonas aeruginosa*								
Piperacillin/tazobactam	30	128	8–≥256	10.0	43	128	4–≥256	39.5
Cefepime	30	≥64	8–≥64	10.0	43	≥64	1–≥64	39.5
Ceftazidime	30	64	4–≥128	16.7	43	32	1–≥128	53.5
Meropenem	30	≥32	1–≥32	16.7	–	–	–	–
Levofloxacin	30	≥16	0.5–≥16	10.0	43	≥16	0.25–≥16	23.3
Amikacin	30	≥128	2–≥128	36.7	43	64	1–≥128	60.5
Tigecycline	30	≥32	0.5–≥32	NA	43	16	≤0.008–≥32	NA

MIC, minimum inhibitory concentration; NA, breakpoints not available; S, susceptible.

-, data not available.

a No isolates were collected in 2014. In addition, there were no data for the following agents in the following years: cefepime, ceftazidime and amikacin in 2019; minocycline in 2018–2021; ampicillin/sulbactam, gentamicin and trimethoprim/sulfamethoxazole in 2010–2017; and imipenem, ciprofloxacin and colistin in 2010–2017 and 2019.

b Some antimicrobial agents were tested against fewer isolates due to fewer years of isolate collection.

#### 3.2.2 *Pseudomonas aeruginosa* (N = 210)

For *P. aeruginosa* isolates from adults, the highest rates of susceptibility were to ceftazidime/avibactam (95.3%), ceftolozane/tazobactam (88.4%) and amikacin (87.1%) (**Table 1**). Data for ceftazidime/avibactam and ceftolozane/tazobactam were not available for the paediatric or ICU subsets, among which susceptibility was highest to amikacin (91.9% and 87.2%, respectively) (**Table 1**). Among isolates from paediatric patients, susceptibility to ceftazidime and piperacillin/tazobactam was 86.5% each, compared with 81.2% and 76.5%, respectively, among isolates from adults. Overall, 30 (14.3%) of *P. aeruginosa* were MDR and 43 (20.5%) were CR. Susceptibility among the MDR isolates was between 10.0% and 36.7% for the available antimicrobials (**Table 2**). Susceptibility was higher among the CR than MDR isolates, with 60.5% of isolate susceptible to amikacin.

### 3.3 Antimicrobial susceptibility of Enterobacterales organisms

#### 3.3.1 *Escherichia coli* (N = 254)

Susceptibility was > 99% to meropenem and tigecycline among the adult and paediatric subsets of *E. coli* (**Table 3**). In addition, susceptibility among isolates from adults was high to ceftazidime/avibactam, ceftolozane/tazobactam, imipenem and meropenem/vaborbactam (94.5%–98.7% [not tested against the paediatric subset]). For *E. coli* isolates from patients in the ICU, all isolates were susceptible to ceftazidime/avibactam, imipenem, meropenem and tigecycline, 85.7% were susceptible to amikacin and 85.0% to gentamicin (**Table 3**).

**Table 3:** *In vitro* activity and antimicrobial susceptibility of the ATLAS antimicrobial panel against clinical isolates of *Escherichia coli* and *Klebsiella pneumoniae* collected in Jordan between 2010 and 2021.

	Adult				Paediatric				ICU (all ages)			
		MIC (mg/L)				MIC (mg/L)				MIC (mg/L)		
Organism^a^/Agent^b^	n	90%	Range	%S	n	90%	Range	%S	n	90%	Range	%S
*Escherichia coli*												
Ampicillin	221	≥64	≤0.5–≥64	12.2	31	≥64	1–≥64	12.9	49	≥64	2–≥64	14.3
Amoxicillin/clavulanate	221	32	0.5–≥64	59.3	31	32	2–≥64	61.3	49	≥64	1–≥64	57.1
Ampicillin/sulbactam	79	64	1–≥128	53.2	–	–	–	–	20	≥128	1–≥128	65.0
Piperacillin/tazobactam	221	32	0.25–≥256	80.5	31	32	0.5–≥256	80.6	49	128	≤0.5–≥256	77.6
Ceftaroline	79	≥16	0.03–≥16	43.0	–	–	–	–	20	≥16	0.03–≥16	50.0
Ceftriaxone	142	64	≤0.06–≥128	44.4	24	≥128	≤0.06–≥128	50.0	29	64	≤0.06–≥128	48.3
Cefepime	221	≥64	≤0.12–≥64	49.3	31	≥64	≤0.12–≥64	48.4	49	≥64	≤0.12–≥64	55.1
Ceftazidime	221	32	0.06–≥256	59.3	31	32	0.12–≥256	54.8	49	32	0.12–≥256	59.2
Ceftazidime/avibactam	79	1	≤0.03–≥128	98.7	–	–	–	–	20	0.25	≤0.03–1	100
Ceftolozane/tazobactam	55	1	0.12–≥32	94.5	–	–	–	–	-	-	-	-
Aztreonam	79	64	≤0.015–≥256	57.0	–	–	–	–	20	32	0.03–≥256	60.0
Aztreonam/avibactam	79	0.5	≤0.015–16	NA	–	–	–	–	20	0.12	≤0.015–0.5	NA
Imipenem	79	1	0.12–≥16	94.9	–	–	–	–	20	0.5	0.12–1	100
Meropenem	221	≤0.06	≤0.06–≥32	99.1	31	≤0.06	≤0.06–0.25	100	49	≤0.06	≤0.06–0.25	100
Meropenem/vaborbactam	55	≤0.06	≤0.06–≥32	98.2	–	–	–	–	-	-	-	-
Ciprofloxacin	79	≥8	≤0.06–≥8	27.8	–	–	–	–	20	≥8	≤0.06–≥8	40.0
Levofloxacin	221	≥16	0.015–≥16	35.7	31	≥16	0.015–≥16	64.5	49	≥16	0.03–≥16	34.7
Amikacin	221	8	0.5–32	88.2	31	4	≤0.5–8	93.5	49	8	1–32	85.7
Gentamicin	79	≥32	0.25–≥32	75.9	–	–	–	–	20	≥32	0.5–≥32	85.0
Colistin	79	1	≤0.12–2	0.0	–	–	–	–	20	0.5	≤0.12–2	0.0
Minocycline	142	≥32	≤0.5–≥32	65.5	24	8	≤0.5–16	87.5	29	≥32	≤0.5–≥32	65.5
Tigecycline	221	0.5	0.03–8	99.1	31	0.5	0.015–0.5	100	49	0.5	0.06–2	100
Trimethoprim/sulfamethoxazole	79	≥64	1–≥64	35.4	–	–	–	–	20	≥64	1–≥64	30.0
*Klebsiella pneumoniae* ^c^												
Amoxicillin/clavulanate	198	32	1–≥64	47.5	50	32	1–≥64	50.0	60	32	1–≥64	31.7
Ampicillin/sulbactam	59	64	1–≥128	32.2	–	–	–	–	27	64	2–≥128	18.5
Piperacillin/tazobactam	198	128	0.12–≥256	62.6	50	128	1–≥256	82.0	60	≥256	0.5–≥256	50.0
Ceftaroline	59	≥16	0.06–≥16	28.8	–	–	–	–	27	≥16	0.06–≥16	18.5
Ceftriaxone	139	64	≤0.06–≥128	46.8	35	64	≤0.06–≥128	48.6	33	64	≤0.06–≥128	30.3
Cefepime	198	≥64	≤0.12–≥64	43.9	50	≥64	≤0.12–≥64	42.0	60	≥64	≤0.12–≥64	26.7
Ceftazidime	198	128	0.06–≥256	42.9	50	64	0.12–≥128	32.0	60	≥128	0.12–≥128	25.0
Ceftazidime/avibactam	59	≥128	0.06–≥128	62.7	–	–	–	–	27	≥128	0.12–≥128	48.1
Ceftolozane/tazobactam	40	≥32	≤0.06–≥32	37.5	–	–	–	–	-	-	-	-
Aztreonam/avibactam	59	0.25	≤0.015–0.25	NA	–	–	–	–	27	0.25	0.03–0.25	NA
Imipenem	59	≥16	0.12–≥16	52.5	–	–	–	–	27	≥16	0.12–≥16	37.0
Meropenem	198	≥32	≤0.06–≥32	81.3	50	0.25	≤0.06–≥32	94.0	60	≥32	≤0.06–≥32	66.7
Meropenem/vaborbactam	40	≥32	≤0.06–≥32	42.5	–	–	–	–	-	-	-	-
Ciprofloxacin	59	≥8	≤0.06–≥8	28.8	–	–	–	–	27	≥8	≤0.06–≥8	14.8
Levofloxacin	198	≥16	≤0.008–≥16	49.5	50	1	0.03–2	82.0	60	≥16	0.03–≥16	38.3
Aztreonam	59	128	≤0.03–128	32.2	–	–	–	–	27	≥128	≤0.03–≥128	18.5
Amikacin	198	16	≤0.5–≥128	78.3	50	8	≤0.5–≥128	88.0	60	≥128	≤0.5–≥128	58.3
Gentamicin	59	≥32	≤0.12–≥32	64.4	–	–	–	–	27	≥32	0.25–≥32	48.1
Colistin	59	2	≤0.12–≥16	0.0	–	–	–	–	27	8	≤0.12–≥16	0.0
Minocycline	139	16	≤0.5–≥32	74.8	35	8	1–16	80.0	33	16	≤0.5–≥32	69.7
Tigecycline	198	2	0.12–8	94.9	50	1	≤0.008–4	98.0	60	1	0.12–8	96.7
Trimethoprim/sulfamethoxazole	59	≥64	1–≥64	45.8	–	–	–	–	27	≥64	1–≥64	33.3

MIC, minimum inhibitory concentration; NA, breakpoints not available; S, susceptible.

-, data not available.

a No isolates were collected in 2014. In addition, there were no data for the following agents in the following years: amoxicillin/clavulanate, cefepime, ceftazidime and amikacin in 2019; minocycline in 2018–2021; ampicillin/sulbactam, ceftaroline, gentamicin and trimethoprim/sulfamethoxazole in 2010–2017; ceftazidime/avibactam, aztreonam, imipenem, ciprofloxacin and colistin in 2010–2017 and 2019; and ceftolozane/tazobactam and meropenem/vaborbactam in 2010–2019.

b Some antimicrobial agents were tested against fewer isolates due to fewer years of isolate collection.

c Ampicillin data not presented due to intrinsic resistance among *K. pneumoniae*.

Compared with the adult subset, a higher percentage of *E. coli* isolates from paediatric patients were susceptible to minocycline (65.5% and 87.5%, respectively) (**Table 3**). Aztreonam/avibactam was not tested against the paediatric subset but the MIC_90_ was 0.5 mg/L against isolates from adults.

More than 90% of MDR *E. coli* were susceptible to ceftazidime/avibactam, ceftolozane/tazobactam, imipenem, meropenem, meropenem/vaborbactam and tigecycline (**Table 4**). Susceptibility was ≥ 80% to meropenem, amikacin and tigecycline among CTX-M-type-positive *E. coli*.

**Table 4:** *In vitro* activity and antimicrobial susceptibility of the ATLAS antimicrobial panel against clinical isolates of *Escherichia coli* and *Klebsiella pneumoniae* with resistance phenotypes and genotypes collected in Jordan between 2010 and 2021.

	Multidrug-resistant					CTX-M-15-positive											
		MIC (mg/L)						MIC (mg/L)									
Organism^a^/Agent^b^	n	90%	Range	%S		n		90%	Range	%S							
*Escherichia coli*																	
Ampicillin	108	≥64	32–≥64	0.0		30		≥64	32–≥64	0.0							
Amoxicillin/clavulanate	108	≥64	2–≥64	32.4		30		32	4–≥64	40.0							
Ampicillin/sulbactam	60	64	2–≥128	40.0		–		–	–	–							
Piperacillin/tazobactam	108	128	0.25–≥256	55.6		30		128	0.5–≥256	60.0							
Ceftaroline	60	≥16	0.03–≥16	18.3		–		–	–	–							
Ceftriaxone	48	≥128	0.12–≥128	20.8		–		–	–	–							
Cefepime	108	≥64	≤0.12–≥64	30.6		30		≥64	2–≥64	3.3							
Ceftazidime	108	64	0.12–≥256	38.0		30		≥256	4–≥256	6.7							
Ceftazidime/avibactam	60	1	≤0.03–≥128	98.3		–		–	–	–							
Ceftolozane/tazobactam	38	1	0.12–≥32	92.1		–		–	–	–							
Aztreonam	60	128	≤0.015–≥256	35.0		–		–	–	–							
Imipenem	60	1	0.12–≥16	93.3		–		–	–	–							
Meropenem	108	0.25	≤0.06–≥32	98.1		30		0.25	≤0.06–≥32	93.3							
Meropenem/vaborbactam	38	0.25	≤0.06–≥32	97.4		–		–	–	–							
Ciprofloxacin	60	≥8	≤0.12–≥8	18.3		–		–	–	–							
Levofloxacin	108	≥16	≤0.25–≥16	19.4		30		≥16	≤0.25–≥16	23.3							
Amikacin	108	8	0.5–32	81.5		30		8	2–32	80.0							
Gentamicin	60	≥32	0.25–≥32	65.0		–		–	–	–							
Colistin	60	0.5	≤0.12–1	0.0		–		–	–	–							
Minocycline	48	≥32	≤0.5–≥32	31.3		–		–	–	–							
Tigecycline	108	0.5	0.06–8	98.1		30		0.5	0.06–8	96.7							
Trimethoprim/sulfamethoxazole	60	≥64	1–≥64	15.0		–		–	–	–							
	Multidrug-resistant					Carbapenem-resistant						CTX-M-15-positive					
		MIC (mg/L)					MIC (mg/L)							MIC (mg/L)			
	n	90%	Range		%S	n	90%		Range		%S	n		90%	Range		%S
*Klebsiella pneumoniae^c^*																	
Amoxicillin/clavulanate	82	≥64	2–≥64		19.5	39	≥64		32–≥64		0.0	74		≥64	4–≥64		17.6
Ampicillin/sulbactam	82	64	8–≥128		5.6	30	64		32–≥128		0.0	41		≥64	8–≥64		4.9
Piperacillin/tazobactam	82	≥256	2–≥256		25.6	39	≥256		128–≥256		0.0	74		128	2–≥256		36.5
Ceftaroline	54	≥16	0.25–≥16		3.7	30	8		8–≥16		0.0	41		≥16	8–≥16		0.0
Ceftriaxone	28	64	≤0.06–≥128		10.7	–	–		–		–	33		≥64	≤0.06–≥64		3.0
Cefepime	82	≥64	≤0.12–≥64		9.8	39	≥64		16–≥64		0.0	74		≥64	4–≥64		0.0
Ceftazidime	82	128	0.12–≥256		7.3	39	≥128		8–≥128		0.0	74		≥128	4–≥128		2.7
Ceftazidime/avibactam	54	≥128	0.12–≥128		53.7	30	≥128		0.5–≥128		16.7	41		≥128	0.12–≥128		39.0
Ceftolozane/tazobactam	37	≥32	0.25–≥32		24.3	27	≥32		≥32		0.0	30		≥32	0.25–≥32		10.0
Aztreonam	54	128	0.03–128		7.4	30	128		32–128		0.0	41		≥128	8–≥128		0.0
Aztreonam/avibactam	54	0.25	≤0.015–0.25		NA	30	0.25		0.06–0.25		NA	41		0.25	≤0.015–0.25		NA
Imipenem	54	≥16	0.12–≥16		40.7	30	≥16		8–≥16		0.0	41		≥16	0.12–≥16		34.1
Meropenem	82	≥32	≤0.06–≥32		51.2	39	≥32		4–≥32		0.0	74		≥32	≤0.06–≥32		56.8
Meropenem/vaborbactam	37	≥32	≤0.06–≥32		29.7	27	≥32		≤0.06–≥32		3.7	30		≥32	≤0.06–≥32		13.3
Ciprofloxacin	54	≥8	≤0.06–≥8		9.3	30	≥8		1–≥8		0.0	41		≥8	≤0.06–≥8		12.2
Levofloxacin	82	≥16	0.06–≥16		26.8	39	≥16		1–≥16		0.0	74		≥16	0.06–≥16		25.7
Amikacin	82	≥128	0.5–≥128		51.2	39	≥128		0.5–≥128		10.3	74		≥128	1–≥128		47.3
Gentamicin	54	≥32	≤0.12–≥32		51.9	30	≥32		0.5–≥32		36.7	41		≥32	0.25–≥32		53.7
Colistin	54	2	≤0.12–≥16		0.0	30	8		≤0.12–≥16		0.0	41		2	≤0.12–≥16		0.0
Minocycline	28	≥32	≤0.5–≥32		53.6	–	–		–		–	33		≥32	1–≥32		72.7
Tigecycline	82	2	0.12–8		92.7	39	1		0.12–1		100	74		2	0.25–8		95.9
Trimethoprim/sulfamethoxazole	54	≥64	1–≥64		29.6	30	≥64		1–≥64		33.3	41		≥64	1–≥64		34.1
	CTX-M-9-type-positive					NDM-5-positive						OXA-48-positive					
		MIC (mg/L)					MIC (mg/L)							MIC (mg/L)			
	n	90%	Range		%S	n	90%		Range		%S	n		90%	Range		%S
*Klebsiella pneumoniae^c^*																	
Amoxicillin/clavulanate	20	≥32	≥32		0.0	24	≥32		≥32		0.0	25		32	32–≥64		0.0
Ampicillin/sulbactam	20	≥128	64–≥128		0.0	24	≥64		32–≥64		0.0	23		≥128	64–≥128		0.0
Piperacillin/tazobactam	20	≥128	≥128		0.0	24	≥128		≥128		0.0	25		128	128–≥256		0.0
Ceftaroline	20	≥16	8–≥16		0.0	24	≥8		≥8		0.0	23		128	32–128		0.0
Cefepime	20	≥64	≥64		0.0	24	≥64		≥64		0.0	23		≥16	8–≥16		0.0
Ceftazidime	20	≥128	8–≥128		0.0	24	≥128		≥128		0.0	25		≥64	2–≥64		4.0
Ceftazidime/avibactam	20	≥128	0.5–≥128		15.0	24	≥128		≥128		0.0	25		128	8–≥256		0.0
Aztreonam	20	128	32–128		0.0	24	≥32		≥32		0.0	23		≥128	0.5–≥128		21.7
Aztreonam/avibactam	20	0.25	0.12–0.25		NA	24	≥128		64–≥128		0.0	23		0.25	0.12–0.25		NA
Imipenem	20	≥16	8–≥16		0.0	24	0.25		0.06–0.25		NA	23		≥16	4–≥16		0.0
Meropenem	20	≥32	16–≥32		0.0	24	≥16		8–≥16		0.0	25		≥32	1–≥32		8.0
Ciprofloxacin	20	≥8	4–≥8		0.0	24	≥32		16–≥32		0.0	23		≥8	2–≥8		0.0
Levofloxacin	20	≥16	8–≥16		0.0	24	≥32		≥32		0.0	25		≥16	2–≥16		0.0
Amikacin	20	≥128	0.5–≥128		15.0	24	≥8		1–≥8		0.0	25		≥128	0.5–≥128		24.0
Gentamicin	20	≥32	0.5–≥32		40.0	24	≥16		1–≥16		0.0	23		≥32	0.25–≥32		39.1
Colistin	20	≥16	≤0.12–≥16		0.0	24	≥128		8–≥128		0.0	23		≥16	≤0.12–≥16		0.0
Tigecycline	20	1	0.25–1		100	24	≥32		0.5–≥32		41.7	25		1	0.25–8		92.0
Trimethoprim/sulfamethoxazole	20	≥64	1–≥64		40.0	24	2		≤0.12–≥16		0.0	23		≥64	1–≥64		43.5

MIC, minimum inhibitory concentration; NA, breakpoints not available; S, susceptible.

-, data not available.

^a^Isolates carrying SHV- or TEM-OSBLs were omitted from this table. Isolates may have carried more than one resistance gene. Organisms presented where ≥20 isolates were reported. No isolates were collected in 2014. In addition, there were no data for the following agents in the following years: amikacin, amoxicillin/clavulanate, cefepime and ceftazidime in 2019; azithromycin in 2019 and 2020; clarithromycin and minocycline in 2018–2021; ampicillin/sulbactam, ceftaroline, daptomycin, gentamicin, teicoplanin and trimethoprim/sulfamethoxazole in 2010–2017; aztreonam, ceftazidime/avibactam, ciprofloxacin, colistin and imipenem in 2010–2017 and 2019; and ceftolozane/tazobactam and meropenem/vaborbactam in 2010–2019. ^b^Some antimicrobial agents were tested against fewer isolates due to fewer years of isolate collection. ^c^Ampicillin data not presented due to intrinsic resistance among *K. pneumoniae*.

Overall, 30 (11.8%) *E. coli* isolates tested positive for CTX-M-15 enzymes, and all were resistant to ampicillin. There were two CR *E. coli* isolates: one of the two isolate was positive for an MBL and the other had no resistance genes identified.

#### 3.3.2 *Klebsiella pneumoniae* (N = 249)

Among isolates from adult and paediatric patients, susceptibility was highest to tigecycline (94.9% and 98.0%, respectively), followed by meropenem (81.3% and 94.0%, respectively) (**Table 3**). Similarly, among isolates from the ICU (all ages), susceptibility was highest to tigecycline (96.7%).

Comparing rates among isolates from paediatrics and adults showed that susceptibility to piperacillin/tazobactam, meropenem, levofloxacin and amikacin was higher among paediatric isolates than those from adults (**Table 3**). Among *K. pneumoniae* from adults, the susceptibility of ceftazidime/avibactam, ceftolozane/tazobactam and meropenem/vaborbactam was 62.7%, 37.5% and 42.5%, respectively, and no isolates were susceptible to colistin.

There were 82 (32.9%) MDR *K. pneumoniae* isolates and 39 (0.2%) CR *K. pneumoniae*. Among MDR *K. pneumoniae* susceptibility was highest to tigecycline (92.7%), followed by ceftazidime/avibactam and minocycline (53.7% and 53.6%, respectively) (**Table 4**). All 39 CR *K. pneumoniae* were susceptible to tigecycline; however, susceptibility was low to the majority of other agents with only 16.7% susceptible to ceftazidime-avibactam, 3.7% to meropenem/vaborbactam, and no isolates susceptible to ceftolozane/tazobactam (**Table 4**). The MIC_90_ for aztreonam/avibactam was 0.25 mg/L. Antimicrobial activity data for *K. pneumoniae* where ≥ 20 isolates had a reported β-lactamase resistance gene are presented in **Table 4**.

Overall, 74 (29.7%) *K. pneumoniae* isolates were positive for CTX-M-15, 20 (8.0%) for CTX-M-9-type, 24 (9.6%) for NDM-5 and 25 (10.0%) for OXA-48-type enzymes. All *K. pneumoniae* with NDM-5 genes were susceptible to tigecycline, as were 92.0% of *K. pneumoniae* carrying OXA-48 genes and ≥ 95.9% carrying CTX-M-type genes. Minocycline susceptibility was 72.7% among *K. pneumoniae* with CTX-M-15 genes (**Table 4**). Among the newer combination agents tested, the MIC_90_ of aztreonam/avibactam was 0.25 mg/L against *K. pneumoniae* with CTX-M-type, NDM-5 and OXA-48 genes. Among the 39 CR *K. pneumoniae* 34 (87.2%) tested positive for a carbapenemase.

### 3.4 Antimicrobial susceptibility of Gram-positive organisms

#### 3.4.1 *Enterococcus faecalis* (N = 122)

*E. faecalis* data were limited to isolates from adults (**Table 5**). All isolates were susceptible to ampicillin, penicillin, teicoplanin, vancomycin, daptomycin and tigecycline, with 98.1% of isolates susceptible to linezolid.

**Table 5:** *In vitro* activity and antimicrobial susceptibility of the ATLAS antimicrobial panel against Gram-positive clinical isolates collected in Jordan between 2010 and 2021.

	Adult				Paediatric				ICU (all ages)			
		MIC (mg/L)				MIC (mg/L)				MIC (mg/L)		
Organism^a^/Agent^b^	n	90%	Range	%S	n	90%	Range	%S	n	90%	Range	%S
*Enterococcus faecalis* ^c^												
Ampicillin	104	1	0.25–2	100	–	–	–	–	-	-	-	-
Penicillin	83	4	1–8	100	–	–	–	–	-	-	-	-
Erythromycin	21	≥8	≤0.12–≥8	9.5	–	–	–	–	-	-	-	-
Teicoplanin	21	0.5	≤0.12–0.5	100	–	–	–	–	-	-	-	-
Vancomycin	104	2	0.5–4	100	–	–	–	–	-	-	-	-
Levofloxacin	104	≥64	0.5–≥64	47.1	–	–	–	–	-	-	-	-
Daptomycin	21	1	0.25–2	100	–	–	–	–	-	-	-	-
Linezolid	104	2	≤0.5–4	98.1	–	–	–	–	-	-	-	-
Minocycline	83	≥16	≤0.25–≥16	25.3	–	–	–	–	-	-	-	-
Tigecycline	104	0.12	≤0.008–0.25	100								
*Staphylococcus aureus*												
Oxacillin	77	≥4	0.25–≥4	49.4	–	–	–	–	20	≥4	0.25–≥4	40.0
Ceftaroline	77	1	0.12–2	96.1	–	–	–	–	20	1	0.25–2	90.0
Erythromycin	77	≥8	0.25–≥8	58.4	–	–	–	–	20	≥8	0.25–≥8	60.0
Clindamycin	77	0.25	0.06–≥4	93.5	–	–	–	–	20	2	0.06–≥4	85.0
Teicoplanin	77	1	≤0.12 to 2	100	–	–	–	–	20	1	≤0.12–2	100
Vancomycin	193	1	0.5–2	100	74	1	0.5–2	100	31	1	0.5–1	100
Levofloxacin	193	8	≤0.06–16	85.0	74	0.5	≤0.06–≥8	95.9	31	≥8	≤0.06–≥8	77.4
Gentamicin	77	≥32	1–≥32	87.0	–	–	–	–	20	≥32	1–≥32	85.0
Daptomycin	77	0.5	0.12–1	100	–	–	–	–	20	0.5	0.12–1	100
Linezolid	193	2	1–4	100	74	2	1–4	100	31	2	1–4	100
Minocycline	116	0.5	≤0.25–≥16	99.1	58	0.5	≤0.25–0.5	100	–	–	–	–
Tigecycline	193	0.25	0.03–0.5	100	74	0.12	0.03–0.5	100	31	0.12	0.03–0.25	100
Trimethoprim/sulfamethoxazole	77	1	≤0.03–≥4	97.4	–	–	–	–	20	0.5	≤0.03–1	100
*Streptococcus pneumoniae*												
Penicillin	48	4	≤0.06–4	16.7	55	4	≤0.06–4	14.5	-	-	-	-
Amoxicillin/clavulanate	43	4	≤0.03–8	83.7	52	4	≤0.03–4	84.6	-	-	-	-
Ceftriaxone	48	1	≤0.015–2	93.8	55	1	≤0.03–2	96.4	-	-	-	-
Meropenem	48	1	≤0.03–2	35.4	55	1	≤0.12–2	40.0	-	-	-	-
Vancomycin	48	0.5	≤0.12–1	100	55	0.5	≤0.12–1	100	-	-	-	-
Azithromycin	43	≥128	0.06–≥128	25.6	52	≥128	≤0.03–≥128	21.2	-	-	-	-
Clarithromycin	43	≥128	≤0.015–≥128	25.6	52	≥128	≤0.015–≥128	21.2	-	-	-	-
Erythromycin	48	≥128	0.03–≥128	25.0	55	64	≤0.015–≥128	18.2	-	-	-	-
Clindamycin	48	≥128	≤0.015–≥128	60.4	55	≥128	≤0.015–≥128	74.5	-	-	-	-
Levofloxacin	48	2	≤0.06–≥8	97.9	55	1	≤0.06–≥8	98.2	-	-	-	-
Linezolid	48	1	≤0.5–1	100	55	1	≤0.5–2	100	-	-	-	-
Tigecycline	48	0.03	≤0.008–0.06	100	55	0.03	0.015–0.06	100	-	-	-	-

MIC, minimum inhibitory concentration; S, susceptible; ICU, intensive care unit.

-, data not available.

a No isolates were collected in 2014. In addition, there were no data for the following agents in the following years: amoxicillin/clavulanate in 2019; azithromycin in 2019 and 2020; minocycline and clarithromycin in 2018–2021; and teicoplanin, gentamicin, ceftaroline, daptomycin and trimethoprim/sulfamethoxazole in 2010–2017; aztreonam, ceftazidime/avibactam, ciprofloxacin, colistin and imipenem in 2010–2017 and 2019; and ceftolozane/tazobactam and meropenem/vaborbactam in 2010–2019.

b Some antimicrobial agents were tested against fewer isolates due to fewer years of isolate collection.

c There were no *E. faecalis* data for the paediatric subset.

#### 3.4.2 *Staphylococcus aureus* (N = 267)

Overall, 157 (58.8%) of *S. aureus* isolates in this study were MRSA. No vancomycin-intermediate or vancomycin-resistant isolates of *S. aureus* from adult or paediatric patients were identified (**Table 5**). In addition, > 99% of *S. aureus* from adult and paediatric patients were susceptible to linezolid, minocycline and tigecycline and. At least 90% of isolates from adults were also susceptible to ceftaroline, clindamycin, teicoplanin, daptomycin, and trimethoprim/sulfamethoxazole (paediatric data not available). Approximately 96% of isolates from paediatric patients were susceptible to levofloxacin, compared with 85.0% of isolates from adults.

All *S. aureus* isolates from the ICU were susceptible to teicoplanin, vancomycin, daptomycin, linezolid, tigecycline and trimethoprim/sulfamethoxazole, and 90.0% were susceptible to ceftaroline (**Table 5**). Similar rates of resistance were seen for MRSA (**Table 6**). In addition, 99.1% of MRSA were susceptible to minocycline.

**Table 6:** *In vitro* activity and antimicrobial susceptibility of the ATLAS antimicrobial panel against clinical isolates of *Staphylococcus aureus* and *Streptococcus pneumoniae* with resistance phenotypes collected in Jordan between 2010 and 2021.

		MIC (mg/L)		
Organism^a^/Agent^b^	n	90%	Range	%S
Methicillin-resistant *S. aureus*				
Ceftaroline	48	1	0.25–2	93.8
Erythromycin	48	≥8	0.25–≥8	39.6
Clindamycin	48	1	0.06–≥4	89.6
Teicoplanin	48	1	0.25–2	100
Vancomycin	157	1	0.5–2	100
Levofloxacin	157	4	≤0.06–≥8	87.3
Gentamicin	48	≥32	1–≥32	87.5
Daptomycin	48	0.5	0.12–1	100
Linezolid	157	2	1–4	100
Minocycline	109	0.5	≤0.25–≥16	99.1
Tigecycline	157	0.25	0.03–0.5	100
Trimethoprim/sulfamethoxazole	48	1	≤0.03–≥4	95.8
Penicillin-resistant *S. pneumoniae*				
Amoxicillin/clavulanate	37	4	0.5–8	59.5
Ceftriaxone	44	2	≤0.03–2	88.6
Meropenem	44	1	0.5–2	0.0
Vancomycin	44	0.5	≤0.12–1	100
Azithromycin	37	≥128	0.12–≥128	8.1
Clarithromycin	37	≥128	0.03–≥128	8.1
Erythromycin	44	≥128	0.06–≥128	6.8
Clindamycin	44	≥128	≤0.015–≥128	56.8
Levofloxacin	44	2	≤0.25–≥8	95.5
Linezolid	44	1	≤0.5–1	100
Tigecycline	44	0.03	0.015–0.06	100

MIC, minimum inhibitory concentration; S, susceptible; ICU, intensive care unit.

a No isolates were collected in 2014. In addition, there were no data for the following agents in the following years: amoxicillin/clavulanate in 2019; azithromycin in 2019 and 2020; minocycline and clarithromycin in 2018–2021; and ceftaroline, teicoplanin, gentamicin, daptomycin and trimethoprim/sulfamethoxazole in 2010–2017.

b Some antimicrobial agents were tested against fewer isolates due to fewer years of isolate collection.

#### 3.4.3 *Streptococcus pneumoniae* (N = 105)

All isolates from adult and paediatric patients were susceptible to vancomycin, linezolid and tigecycline (**Table 5**). There were also high rates of susceptibility to ceftriaxone and levofloxacin among both adult and paediatric groups (93.8%–98.2%). No *S. pneumoniae* isolates were collected from ICUs. All penicillin-resistant *S. pneumoniae* were susceptible to vancomycin, linezolid, tigecycline with 95.5% and 88.6% susceptible to levofloxacin and ceftaroline, respectively (**Table 6**). All penicillin-resistant *S. pneumoniae* isolates were resistant to meropenem.

## 4 Discussion

This study of bacterial isolates collected from Jordan between 2010 and 2021 found that susceptibility to ceftazidime/avibactam and ceftolozane/tazobactam was high among *E. coli*, and *P. aeruginosa* collected from adult patients (> 88%), and was also high to meropenem and meropenem/vaborbactam among *E. coli* from adults (> 97%). The highest susceptibility among *K. pneumoniae* isolates from adult patients was to tigecycline (94.9%). Where data were available, similar results to those reported for adults were seen for isolates from paediatric patients and for patients from the ICU, although there was more variation in susceptibility between the different subsets for isolates of *A. baumannii* and *K. pneumoniae*. The analysis of MDR and CR Gram-negative isolates demonstrated the lack of activity of many agents against these difficult to treat organisms. In addition, antimicrobial susceptibility among isolates of *E. coli* and *K. pneumoniae* carrying β-lactamase genes to most agents, was ≤ 60.0%. Ceftaroline susceptibility was ≥ 90.0% against *S. aureus*, including MRSA isolates and all Gram-positive isolates collected as part of this study in Jordan were susceptible to tigecycline.

There are limited published antimicrobial surveillance data from Jordan on novel agents, such as ceftazidime/avibactam, ceftolozane/tazobactam, meropenem/vaborbactam and ceftaroline. A multi-hospital study of MDR Gram-negative isolates from Jordan reported susceptibility rates of 73% for ceftazidime/avibactam and 62% for ceftolozane/tazobactam among CR *P. aeruginosa* collected between 2019 and 2020 (Al Ramahi et al., 2021). Higher susceptibility rates for ceftazidime/avibactam and ceftolozane/tazobactam were reported in the current study against *P. aeruginosa* from adults (95.3% and 88.4%, respectively); however, this was for *P. aeruginosa* irrespective of carbapenem activity. Only two isolates of *P. aeruginosa* among the 210 collected in the present study were found to carry β-lactamase genes [1 isolate carrying VIM (type not recorded) and 1 isolate carrying VIM-4 (**Supplemental Data**, **Supplementary Table 2**)], indicating that *P. aeruginosa* from these patients had not acquired a high proportion of resistance genes during the study period. A 2017–2020 SMART study found that 81.1% of all *P. aeruginosa* isolates from Jordan were susceptible to ceftolozane/tazobactam, in agreement with the current study (Lob et al., 2022). Similar to the present study data, a 2015–2018 study of Gram-positive isolates from skin and soft tissue infections or respiratory tract infections reported a ceftaroline MIC of < 2 mg/L for all *S. aureus*, (including MRSA) (Karlowsky et al., 2021b).

The suggested emerging pathogen threats to paediatric patients are MRSA, vancomycin-resistant *S. aureus*, and ESBL-producing and CR Enterobacterales (Romandini et al., 2021). In the current study, ≥ 94.0% of *E. coli* and *K. pneumoniae* from paediatric patients were susceptible to meropenem, and all *S. aureus* were susceptible to vancomycin. Among the newer agents tested, paediatric data were not available for ceftazidime/avibactam or ceftaroline, which are active against the aforementioned emerging pathogens and approved for paediatric infections. A 2012–2019 surveillance study of isolates from paediatric patients in Kuwait showed that most Enterobacterales (including resistance phenotypes) and *P. aeruginosa* were susceptible to ceftazidime/avibactam, and most Gram-positive isolates, including resistance phenotypes, were susceptible to ceftaroline (Al-Sweih et al., 2021). Thus, ceftazidime/avibactam and ceftaroline represent promising options for paediatric infections, including those caused by antimicrobial-resistant organisms.

In the present study, the most frequent β-lactamase gene identified among *E. coli* and *K. pneumoniae* isolates from Jordan was CTX-M-15. CTX-M-15 was also the most frequent ESBL reported in previous studies from Kuwait, Jordan and Lebanon (Al-Sweih et al., 2021; Hayajneh et al., 2015; Moubareck et al., 2005). Meropenem. amikacin and tigecycline were active against CTX-M-15-positive *E. coli* (80.0%–96.7% susceptible) in the current study, while tigecycline was also active against *K. pneumoniae* isolates harbouring CTX-M-15 (95.9% susceptible). In contrast, the susceptibility of CTX-M-15-positive *K. pneumoniae* to ceftazidime/avibactam in the present study was ≤ 39.0%. Research has shown that the CTX-M-15 enzyme purified from *E. coli* demonstrated an increased catalytic activity against ceftazidime than other types of CTX-M ESBLs (Poirel et al., 2002), which could explain the low susceptibility to ceftazidime/avibactam.

Overall, 15.7% of *K. pneumoniae* and 0.8% of *E. coli* (39 and 2 isolates, respectively) were CR. Carbapenem-resistance would appear to be becoming an increasing problem in Jordan with the CR *K. pneumoniae* in this study emerging since 2015 (data not shown) and the *E. coli* collected in 2020 and 2021. The majority of these isolates were carbapenemase-positive (35/41, 85.4%); however, 6 isolates (5 K*. pneumoniae*, 1 *E. coli*) were not carbapenemase-positive, suggesting that carbapenem-resistance was mediated through other mechanisms in this small number of isolates. Alternative mechanisms of resistance can be either porin-mediated or through efflux pumps (Elshamy and Aboshanab, 2020). The ATLAS programme does not have methodology in place to detect such mechanisms and this word be a beneficial addition to the programme as CR isolates continue to develop and pose a challenge to effective antimicrobial treatment.

Of concern is the marked lack of antimicrobial activity against *K. pneumoniae* that were NDM-5-positive (100% resistance or nonsusceptibility to 84% of tested agents with breakpoints), CTX-M-9-type-positive (100% resistance or nonsusceptibility to 71% of agents with breakpoints), and OXA-48-positive (100% resistance or nonsusceptibility to 59% of tested agents with breakpoints). *K. pneumoniae* carrying CTX-M-9-type genes tended to exhibit lower rates of antimicrobial susceptibility to the majority of agents, except for tigecycline and trimethoprim/sulfamethoxazole, than isolates that were CTX-M-15-positive. A 2012–2013 study of *K. pneumoniae* in Jordan found that, among carbapenemase-producers CTX-M-9 was harboured by both NDM- and OXA-producers (Aqel et al., 2017). Although CTX-M-9 enzymes have demonstrated higher catalytic activity against cefotaxime than ceftazidime, compared with other tested CTX-M-type enzymes (Bonnet et al., 2001), the co-carriage of CTX-M-9 with OXA-48-like or NDM-5 genes would appear to result in a loss of antimicrobial activity for most agents, with only tigecycline active against all of these subsets. The MIC_90_ of aztreonam/avibactam was 0.25 mg/L against each subset of β-lactamase-positive *K. pneumoniae* (versus aztreonam MIC_90_ values of 128–≥ 128 mg/L), similar to previous global surveillance studies (Biedenbach et al., 2015; Esposito et al., 2021). To date, clinical data have shown that aztreonam/avibactam has been effective and well-tolerated, with a similar safety profile to aztreonam alone (Pfizer Inc, 2023). This agent could therefore represent a valuable future treatment option. In addition, as MBL-producing pathogens are often also MDR through the co-production of other β-lactamases, such as ESBLs, ceftazidime/avibactam may be used in combination with aztreonam in clinical practice to target a greater variety of β-lactamases than each agent alone (Falcone et al., 2021).

We recognise a number of limitations with the ATLAS programme and our analysis. Although the ATLAS surveillance programme is a valuable tool for the monitoring and tracking of antimicrobial activity, the study protocol defines the number of isolates from each species that can be submitted by each centre, each year; therefore, the study cannot be considered to assess the prevalence of antimicrobial-resistant bacteria. The yearly participation of each medical centre in Jordan varied (with no centres participating in 2014) and there was some variation in the number of isolates tested against each antimicrobial agent by year, because of changes in the antimicrobial testing panel, which prevented a year-by-year analysis. In addition, as not all isolates that met the criteria for molecular testing in 2020 and 2021 were tested, there are a small number of isolates which may have carried β-lactamase genes that were not identified. As with all surveillance studies, caution should be exercised in extrapolating surveillance data; nevertheless, the data we report present a picture of the antimicrobial susceptibilities among clinical isolates from adult and paediatric patients in Jordan.

In summary, *A. baumannii* presents considerable challenges to treatment because of the high percentages of MDR and CR (> 90% of isolates). In contrast, most of the tested *P. aeruginosa* isolates collected from patients in Jordan were susceptible to ceftazidime/avibactam and ceftolozane/tazobactam. Aztreonam/avibactam was active against isolates of *E. coli* and *K. pneumoniae*, including β-lactamase-positive *K. pneumoniae*, although no breakpoints are currently available to determine susceptibility. Tigecycline was active against most Gram-negative and all Gram-positive isolates from adult and paediatric patients, and the majority of *S. aureus* isolates were susceptible to ceftaroline. In additional to tigecycline, all *S. aureus* were susceptible to teicoplanin, vancomycin, daptomycin and linezolid and all *S. pneumoniae* were susceptible to vancomycin, linezolid and tigecycline. CR and MDR Gram-negative isolates exhibited susceptibility rates of < 60% to most antimicrobial agents. CTX-M-15 was the most frequently identified clinically significant β-lactamase gene among *E. coli* and *K. pneumoniae*, which exhibited reduced susceptibility to most agents. In conclusion, the data presented here offer vital information to clinicians and infectious disease specialists to allow them to make informed decisions on appropriate antimicrobial prescribing, based on antimicrobial activity.

## Data availability statement

The original contributions presented in the study are publicly available. This data can be found here: NCBI RefSeq - resistance gene CTX-M-15, accession NG_048935.1; resistance gene CTX-M-9, accession NG_049043.1; resistance gene OXA-48, accession NG_049762.1; resistance gene NDM-5, accession NG_049337.1.

## Ethics statement

Ethical approval was not required for the study involving humans in accordance with the local legislation and institutional requirements. Written informed consent to participate in this study was not required from the participants or the participants’ legal guardians/next of kin in accordance with the national legislation and the institutional requirements.

## Author contributions

DA: Writing – original draft, Writing – review & editing. JB: Writing – original draft, Writing – review & editing. JM: Writing – original draft, Writing – review & editing. JA: Writing – original draft, Writing – review & editing.
