# Supplementary material for: In vitro antimicrobial susceptibility of clinical isolates from adult and paediatric patients in Jordan: Antimicrobial Testing Leadership and Surveillance (ATLAS) 2010–2021
Source: Front Antibiot. 2024 Aug 8;3:1375980. doi: 10.3389/frabi.2024.1375980 (PMC11732154; doi:10.3389/frabi.2024.1375980)
Supplement: Supplementary file 1 [file Table_1.docx]

# Supplementary Table 1. Demographic information for Gram-negative and Gram-positive clinical isolates from Jordan (ATLAS, 2010–2021^a^)

| **Demographic category^b^** | **Isolates, n (%)** | | |
| --- | --- | --- | --- |
|  | **Gram-negative, 1,248 (67.2%)** | **Gram-positive, 608 (32.8%)** | **Total, 1,856** |
| **Patient sex** |  |  |  |
| Female | 595 (47.7%) | 311 (51.2%) | 906 (48.8%) |
| Male | 653 (52.3%) | 297 (48.8%) | 950 (51.2%) |
| **Patient age (years)** |  |  |  |
| 0–17 | 251 (20.1%) | 160 (26.3%) | 411 (22.1%) |
| ≥18 | 997 (79.9%) | 448 (73.7%) | 1445 (77.9%) |
| **Source of infection** |  |  |  |
| Circulatory | 226 (17.8%) | 142 (23.2%) | 368 (19.6%) |
| Genitourinary | 316 (24.9%) | 170 (27.7%) | 486 (25.8%) |
| HEENT | 56 (4.4%) | 39 (6.4%) | 95 (5.1%) |
| Instruments | 7 (0.6%) | 0 (0%) | 7 (0.4%) |
| Intestinal | 179 (14.1%) | 109 (17.8%) | 288 (15.3%) |
| Nervous System | 10 (0.8%) | 3 (0.5%) | 13 (0.7%) |
| Respiratory | 249 (19.6%) | 50 (8.2%) | 299 (15.9%) |
| Skin / Musculoskeletal | 222 (17.5%) | 97 (15.8%) | 319 (17.0%) |
| None Given | 3 (0.2%) | 3 (0.5%) | 6 (0.3%) |
| **Hospital Ward** |  |  |  |
| Clinic / Office | 157 (12.4%) | 119 (19.4%) | 276 (14.7%) |
| Emergency Room | 155 (12.2%) | 81 (13.2%) | 236 (12.5%) |
| General Unspecified ICU | 17 (1.3%) | 3 (0.5%) | 20 (1.1%) |
| Medicine General | 329 (25.9%) | 136 (22.2%) | 465 (24.7%) |
| Medicine ICU | 141 (11.1%) | 30 (4.9%) | 171 (9.1%) |
| Nursing Home / Rehab | 5 (0.4%) | 1 (0.2%) | 6 (0.3%) |
| Other | 1 (0.1%) | 1 (0.2%) | 2 (0.1%) |
| Paediatric General | 58 (4.6%) | 55 (9.0%) | 113 (6.0%) |
| Paediatric ICU | 41 (3.2%) | 15 (2.4%) | 56 (3.0%) |
| Surgery General | 220 (17.4%) | 119 (19.4%) | 339 (18.0%) |
| Surgery ICU | 83 (6.5%) | 18 (2.9%) | 101 (5.4%) |
| None Given | 61 (4.8%) | 35 (5.7%) | 96 (5.1%) |
| **Organism^c^** (*n*) |  |  |  |
|  | *A. baumannii* (160) | *E. faecalis* (122) |  |
|  | *P. aeruginosa* (210) | *S. aureus* (267) |  |
|  | *E. coli* (254) | *S. pneumoniae* (105) | - |
|  | *K. pneumoniae* (249) | *S. agalactiae* (82) | - |
|  | *E. cloacae* (148) | *E. faecium* (20) | - |
|  | *S. marcescens* (69) |  | - |
|  | *K. aerogenes* (21) |  |  |
|  | *H. influenzae* (85) |  |  |

HEENT, head, ears, eyes, nose and throat; ICU, intensive care unit.

^a^No isolates were collected in 2014.

^b^Some patients did not have fully completed records. In addition, isolates may have come from >1 source of infection or hospital ward.

^c^Isolate numbers varied by antimicrobial agents tested, with the maximum numbers of tested isolates presented here. Not all species collected are listed.

# Supplementary Table 2. Counts of resistance genotype frequency among Gram-negative clinical isolates from Jordan (ATLAS, 2010–2021^a^)

| Organism | Genotype | Frequency^b^ | %^c^ |
| --- | --- | --- | --- |
| *Klebsiella pneumoniae* | SHV-OSBL | 80 | 21.1 |
| *Klebsiella pneumoniae* | CTX-M-15 | 74 | 19.5 |
| *Klebsiella pneumoniae* | TEM-OSBL | 62 | 16.4 |
| *Escherichia coli* | CTX-M-15 | 30 | 7.9 |
| *Klebsiella pneumoniae* | OXA-48 | 25 | 6.6 |
| *Klebsiella pneumoniae* | NDM-5 | 24 | 6.3 |
| *Escherichia coli* | TEM-OSBL | 20 | 5.3 |
| *Klebsiella pneumoniae* | CTX-M-9-TYPE | 20 | 5.3 |
| *Escherichia coli* | CTX-M-27 | 5 | 1.3 |
| *Klebsiella pneumoniae* | NDM-1 | 5 | 1.3 |
| *Klebsiella pneumoniae* | OXA-181 | 5 | 1.3 |
| *Escherichia coli* | DHA-1 | 3 | 0.8 |
| *Klebsiella pneumoniae* | DHA-1 | 3 | 0.8 |
| *Klebsiella pneumoniae* | DHA-TRUNC | 3 | 0.8 |
| *Escherichia coli* | CTX-M-9-TYPE | 2 | 0.5 |
| *Escherichia coli* | SHV-12 | 2 | 0.5 |
| *Klebsiella pneumoniae* | CTX-M-14 | 2 | 0.5 |
| *Klebsiella pneumoniae* | KPC-2 | 2 | 0.5 |
| *Escherichia coli* | CMY-16 | 1 | 0.3 |
| *Escherichia coli* | CMY-4 | 1 | 0.3 |
| *Escherichia coli* | CMY-TYPE | 1 | 0.3 |
| *Escherichia coli* | CTX-M-143 | 1 | 0.3 |
| *Escherichia coli* | NDM-5 | 1 | 0.3 |
| *Escherichia coli* | SHV-OSBL | 1 | 0.3 |
| *Klebsiella aerogenes* | OXA-48 | 1 | 0.3 |
| *Klebsiella pneumoniae* | SHV-11 | 1 | 0.3 |
| *Klebsiella pneumoniae* | SHV-2 | 1 | 0.3 |
| *Klebsiella pneumoniae* | SHV-ESBL | 1 | 0.3 |
| *Pseudomonas aeruginosa* | VIM | 1 | 0.3 |
| *Pseudomonas aeruginosa* | VIM-4 | 1 | 0.3 |

^a^No isolates were collected in 2014.

^b^Individual isolates may have carried more than one resistance gene.

^c^% of total isolates presented in table (*n* = 379)
